# Design and characterization of plasticized bacterial cellulose/waterborne polyurethane composite with antibacterial function for nasal stenting

**DOI:** 10.1093/rb/rbaa029

**Published:** 2020-10-15

**Authors:** Zhaoxuan Feng, Minglu Li, Xing Jin, Yudong Zheng, Junxiu Liu, Liang Zhao, Yansen Wang, Hao Li, Danlin Zuo

**Affiliations:** 1 School of Material Science and Engineering, University of Science and Technology Beijing, Beijing, China; 2 Department of Otorhinolaryngology, Peking University Third Hospital, Beijing, China; 3 Research Center for Bioengineering and Sensing Technology, University of Science and Technology Beijing, Beijing 100083, China; 4 School of Chemistry and Biological Engineering, University of Science and Technology Beijing, Beijing 100083, China

**Keywords:** bacterial cellulose, polyurethane, biocompatibility, nasal stent

## Abstract

A nasal stent capable of preventing adhesions and inflammation is of great value in treating nasal diseases. In order to solve the problems of tissue adhesion and inflammation response, we prepared plasticized bacterial cellulose (BCG) and waterborne polyurethane (WPU) composite with antibacterial function used as a novel nasal stent. The gelation behavior of BCG could contribute to protecting the paranasal sinus mucosa; meanwhile, the WPU with improved mechanical property was aimed at supporting the narrow nasal cavity. The thickness, size and the supporting force of the nasal stent could be adjusted according to the specific conditions of the nasal. Thermogravimetric analysis, contact angle and water absorption test were applied to investigate the thermal, hydrophilic and water absorption properties of the composite materials. The composite materials loaded with poly(hexamethylene biguanide) hydrochloride maintained well antibacterial activity over 12 days. Animal experiments further revealed that the mucosal epithelium mucosae damage of BCG−WPU composite was minor compared with that of WPU. This new type of drug-loaded nasal stent can effectively address the postoperative adhesions and infections while ensuring the health of nasal mucosal, and thus has an immense clinical application prospects in treating nasal diseases.

## Introduction

Chronic rhinosinusitis (CRS) and other nasal structural abnormalities are the most frequently occurring diseases in otolaryngology. They are prone to cause salivation, nasal congestion, head pain and even dysosmia, which have adverse effects on the living life of most patients [1−3]. Functional endoscopic sinus surgery (FESS) combined with nasal spray [4] or antibiotics drugs is currently the preferred treatment [5, 6]. However, this surgery has a high recurrence rate [7−12]. Sinus stent is considered to be effective in reducing postoperative adhesions, infections and recurrence of nasal polyps, and is an adequate method to improve the therapeutic benefits of FESS [13−18].

In recent years, researches toward sinus stent have made breakthroughs in material selection, stent morphology design, drug release [19−21] and biodegradability [22, 23], which greatly improving the treatment experience of patients. Eifler *et al*. [7] prepared a biodegradable and biocompatible Mg−Nd alloy stent. *In vitro* cell culture of primary pig nasal epithelial cells showed good biocompatibility of the alloy, and *in vivo* implantation showed no indication of systemic or local side-effects that were generated by employing the alloy. However, only 63% of volume loss was observed after a long-term implantation period of 180 days. Murr *et al*. demonstrated the safety and efficacy of a polylactic acid (PLA) steroid-eluting stent for CRS patients. The follow-up results showed that the stent was effective in wound healing by preserving sinus patency, reducing inflammation and minimizing adhesions via controllable local steroid delivery. Although the biodegradable metallic and hard polymer materials have good biocompatibility and obvious supporting effect, its strong strength and hardness may cause mucosa scratch, and the patients may suffer severe foreign body sensation at the implantation site. Also, *in vivo* data have shown that the imbedding of hard materials into animal frontal sinus ostium had obvious fibrosis and osteogenesis, but little or no epithelialization [24]. Therefore, it is particularly important to analyze the internal structure of the nasal cavity and select the appropriate material to individually design a nasal stent with adjustable shape, size, thickness and supporting force according to the structure and size of the nasal cavity.

Waterborne polyurethane (WPU) has excellent properties such as non-toxicity, satisfactory mechanical performance and good biocompatibility [19, 25, 26], and is widely used in wound dressings, tissue engineering [27, 28] and other biomedical fields [29, 30]. Bacterial cellulose (BC) has always been a popular material for wound dressings and substituted skin due to its good water absorption property, excellent biocompatibility, high permeability and high Young’s modulus [31−35]. Qiao *et al*. [36] pointed out that the modified BC can promote the migration of endothelial cells and fibroblasts. In addition, researches have reported that BC could provide a good local environment for wound and actively promote wound healing [37−39]. Actually, the ideal nasal stent material should not only expand the nasal cavity to prevent adhesion and ensure nasal ventilation, but also effectively promote wound healing and epithelialization.

**Figure 1:**
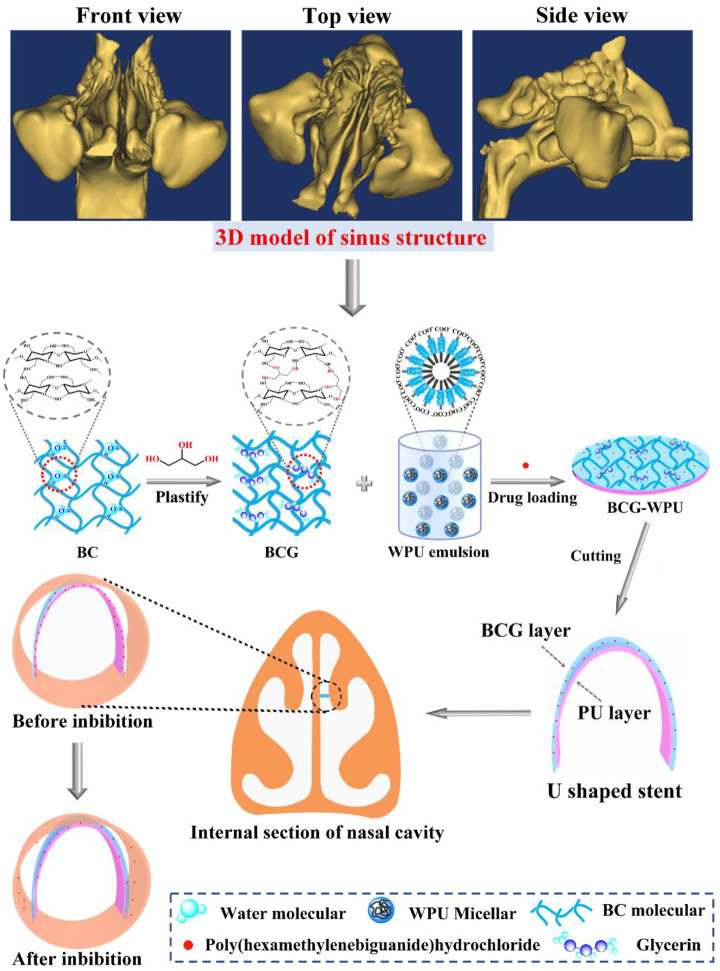
Schematic representation of the design idea and the fabrication process of the BCG−WPU composite drug-loaded nasal stent

In this study, plasticized bacterial cellulose (BCG) and WPU composite with antibacterial activity for nasal stenting was first designed and prepared based on the exact size and structure of the sinus obtained by Materialise’s interactive medical image control system (MIMICS). In our design (Fig. 1), the WPU emulsion infiltrated the plasticized bacterial cellulose and combined to form a two-layer structure through hydrogen bonding. The WPU could hold the cavity of sinus to ensure good nasal ventilation, while the BCG could enhance the supporting force of WPU, and its gel state after absorbing water may also protect nasal mucosa and reduce the discomfort of stent imbedding. In order to validate the performance and realizability of the composite nasal stent we designed, the hydrophilicity, water absorption properties, tensile mechanical properties and supporting strength of the composite materials were successively studied and analyzed combining with the internal structure and physiologic factor. Considering the complex environment and physiologic factor in the nasal cavity after surgery, we endowed the designed nasal stent with antimicrobial activity by loading poly(hexamethylene biguanide) hydrochloride (PHMB) on the composite materials, and the long-term antimicrobial effect of the composite materials was subsequently evaluated. Finally, the *in vitro* and the *in vivo* biological performance of the composite material were evaluated through cell compatibility experiments and rabbit nasal cavity implantation experiments.

## Materials and Methods 

### Materials

Bacterial cellulose (BC) was purchased from the Hainan Yida Food Co., Ltd. The glycerol [polyethylene glycol (PEG)] was purchased from Sinopharm Chemical Reagent Co., Ltd. PHMB was purchased from Shanghai Yuanye Biotechnology Co., Ltd. All the other reagents were analytical grade.

### Construction of 3D model of nasal sinus structure

A healthy adult male face CT scanned image was selected and imported into the medical image processing software (Mimics 17.0, Materialise, Belgium). Erase, draw and other editing commands were used to check and fill the contour lines of the scanned images of each layer of the nasal sinuses. Through thresholding, editing masks and other operations, the boundaries of the human facial sinus cavity, the nasal airway, the surrounding soft tissue and bone tissue were completely separated. The above operation steps were repeated until a completely satisfactory mask was obtained. Finally, the editing toolbar was opened and the 3D model of the nasal sinus was established by clicking the calculate 3D button. The 3D model of the nasal sinus could be observed in multiple directions through the 3D view button.

### Preparation of BCG−WPU stent loaded with PHMB

The bacterial cellulose membranes with average thickness of 3 mm, was treated with aqueous solution of NaOH (0.1 M) at 90°C for 1 h to remove the bacterial debris and impurities, and then it was thoroughly washed to neutral with deionized water [32]. The cleaned bacterial cellulose was submitted to a solvent exchange process in glycerol to get better flexibility and strength, and the plasticized bacterial cellulose obtained in this step is named BCG. BCG membranes were cut into round sheets with 9 cm diameter and pressed between two polyterafluoroethylene plates under the applied pressure of 500 kPa. The flattened bacterial cellulose was immersed in a mixed aqueous solution of glycerol and PHMB for 24 h, and then the plasticized bacterial cellulose (BCG) loaded with PHMB was obtained.

The WPU was synthesized previously referring to the literature [6] by a three-step polymerization procedure. First, PEG, polybutylene glycol adipate and isophorone diisocyanate were placed into a reaction flask for prepolymerization at the presence of the catalyst stannous octanoate. Dimethylolpropionic acid, as the hydrophilic chain-extender, was then added to the flask for chain extension. After neutralized with trimethylamine, the deionized aqueous solution of l-arginine was added and stirred vigorously to carry out an emulsification reaction.

The WPU emulsion loaded with PHMB was casted on the top of BC and BCG sheets in an annular mold. The composite films were dried in a vacuum oven at 37°C for 24 h, and then BC−WPU and BCG−WPU stent loaded with PHMB were obtained by clipping and smoothing the composite films into different size.

### Characterization of the stent materials

#### Surface and interface morphology

The morphology of surfaces and microstructures, interfaces and cross-sections of composite materials was observed by scanning electron microscope (SEM) (FE-SEM, Zeiss Supra) at 5 kV. BC and BCG membranes were freeze-dried. All the samples were sputtered with carbon before observation by SEM to improve the image resolution. 

#### Thermal property

Samples of ∼10 mg were analyzed by thermogravimetric analysis (TGA) (Beijing Evergreen Instruments Co., Ltd). The temperature of sample chamber was raised from room temperature to 600°C. Nitrogen was used to protect the atmosphere. The heating rate was 10°C/min, and the flow rate of nitrogen was 50 ml/min.

#### Water absorption capacity

BC−WPU, BCG−WPU and WPU films were cut into disk shapes with 1.5 cm diameter, initial masses (*W*_0_) measured. The samples then were immersed in deionized water to make sure a complete absorption at room temperature. At different time points, the surface water of the samples was wiped off, and the wet mass (*W*_t_) was recorded. The water absorption rate (*Q*) was calculated as follow:

(1) $$Q=\frac{W_{t}-W_{0}}{W_{t}}\times 100\%$$

#### Hydrophilicity

The water contact angle (WCA) of BC, BCG, WPU, BC−WPU and BCG−WPU films was tested on a high definition video contact angle measuring instrument (Kruss, Germany). The WCA was measured at room temperature using 2 μl drops of distilled water.

### Mechanical properties

The dumbbell-shaped samples (*n* > 4) were prepared by cutting the BC, BCG, WPU, BC−WPU and BCG−WPU films with a custom steel cutter according to ISO standards [ISO 37:2005(E)]. Mechanical testing was performed with dumbbell-shaped samples on the TA-HD plus Texture Analyzer (Stable Micro Systems Co. Ltd, UK) at a drawing speed of 1 mm/min. The Young’s modulus was calculated as the slope at the initial linear region.

### Supporting force of U-shaped stent

BCG−WPU samples (1 × 2 cm) with different thickness of 0.6, 0.7, 0.8, 1.0 and 1.3 cm to test the U shape supporting force on the TA-HD plus Texture Analyzer (Stable Micro Systems Co. Ltd) at a compress speed of 1 mm/min. We also prepared WPU, BC−WPU, BCG−WPU (1 × 2 cm rectangle) with the thickness of 1.3 mm to test the effect of compositing BC or BCG on the supporting force of WPU. The test method was the same as above.

### Antibacterial performance of BCG−WPU stent

Gram-negative *Escherichia coli* (*E. coli*, ATC25922) and Gram-positive *Staphylococcus aureus* (*S. aureus*, ATC25923) were used for bacteriostatic tests. On a sterile test bench, standard strains were placed on MacConkey platelets and incubated for 24 h at 37°C. The grown colonies were diluted with sterile saline and centrifuged for 20 − 30 min at 2500 − 3000 rpm. After centrifugation, the colony density was adjusted to 10^5^ CFU/ml. *Staphylococcus aureus* and *E. coli* of densities of 10^5^ CFU/ml were diluted 1:100 and plated on Luria-Bertani agar plates. All samples were sterilized in a high temperature autoclave for 30 min at 120°C before testing. The sterilized sample was placed in the center of the agar plate, and appropriately squeezed to make a close contact with the agar plate base. Finally, the agar plates were placed in a constant temperature and humidity incubator at 37°C for 24 h. After the bacteria were evenly grown, the agar plates were taken out, and the diameter of the inhibition zone was measured.

The antibacterial effect of BCG and WPU loaded with PHMB at different times was tested as follows: BCG and WPU loaded with PHMB samples were put into test tubes, and 3 ml sterilized PBS solution was added. The samples were vibrated in a constant temperature oscillator, and the extracts were absorbed and preserved at different times; for three test tubes, each of tubes was added 1 ml of PBS, 1.5 ml of culture medium and 1 ml of *E. coli* solution as control group; for the experimental groups, 1 ml of extract of the test sample oscillated at specific time, 1.5 ml of culture medium and 1 ml of *E. coli* solution were added to each test tube; all the tubes were sealed and place in a constant temperature oscillator for 24 h at 150 rpm; The OD value of the liquid of each tube was measured by a ultraviolet spectrophotometer at 600 nm.

### *In vitro* cytocompatibility

The cytotoxicity test was carried out in accordance with ‘GB/T 16886.5-2003 Biological Evaluation of Medical Devices Part 5: In vitro Cytotoxicity Test’. The freeze-dried BC, BCG and WPU samples were irradiated with ultraviolet light for 60 min, and then placed in a sterile centrifuge tube. Fresh cell culture medium was added (6 cm^2^/ml) and the tubes were then place in a carbon dioxide incubator. After 24 h, the extract was aspirated and placed in a new 15 ml sterile centrifuge tube. Rat fibroblasts (L929s) were aspirated to the original culture solution, and then digested with 0.25% trypsin. After ∼3 min, the prepared culture solution was added to terminate the digestion. The cells were uniformly dispersed in the culture solution by pipetting repeatedly. The cells were then seeded on a 96-well plate at a density of 3 × 10^4^ cells/ml. The prepared extract was added after the cells were completely attached on the bottom of the plate. On Days 1, 3 and 7 after incubation, 90 μl of serum-free DMEM medium and 10 μl of CCK-8 reagent were added. After incubation at 37°C for 4 h, the absorbance OD value was measured at 450 nm on a microplate reader.

Cell compatibility was also evaluated by inoculating cells on BC, BCG and WPU to observe cell adhesion and proliferation on these materials. In brief, the dried BC, BCG and WPU samples (*n* = 9) were irradiated with ultraviolet light for 60 min and placed in 24-well plate; L929 cells were digested with trypsin and inoculated on the samples at a concentration of 5 × 10^4^; the samples were washed with PBS at the 1, 3 and 7 days of culture, and then stained with Calcein-AM to observing the cell adhesion and proliferation on an inverted fluorescence microscope.

### *In vivo* performance

The Japanese white rabbits with average weight of 4.2 − 5.5 kg used in this study were purchased and fed at the Department of Laboratory Animal Science of Peking University Health Science Center. Animal ethical committee study No. LA2018250 (approval date 27 June 2018) with approval from Peking University biomedical ethics committee. BCG−WPU and WPU were used as experimental groups, and the non-implant groups were used as the blank control of *in vivo* performance, and BC−WPU was excluded because of its poor mechanical properties. The samples were cut into 1.2 × 0.5 mm sheets with thickness of 0.7 mm. Twelve rabbits were randomly divided into WPU group and BCG−WPU group, six of them were implanted with WPU on one side with blank on the other; six of them were randomly implanted with BCG−WPU on one side with blank on the other. Approximately 30 min before the procedure, the rabbits were anesthetized with ketamine (50 mg/kg). Use a gun forcep to expand the nasal cavity of the rabbit and place the stents into the mucosa of the inferior turbinate. The samples were fixed with 5 − 0 Vicryl suture (Ethicon, Inc., Somerville, NJ, USA). The rabbits received injection of enrofloxacin (5 mg/kg, SQ) once daily for 2 days post-operatively. Six rabbits were sacrificed using a phenobarbital overdose on the third and seventh day after implantation of the stent, respectively. Then the nasal part of rabbit was skinned and removed. The inferior turbinates were gathered and fixed in 10% neutral buffered formalin for 24 h, then embedded tissues with paraffin; paraffin section, xylene dewaxing and hematoxylin and eosin staining; Then gradient alcohol (60, 75, 85, 95, 100%) and xylene were used to dehydration; the histological images were observed on a biomicroscope (Leica DM500, Germany) Specimens of BCG−WPU and WPU were removed and washed by PBS, and soaked in 4% paraformaldehyde solution for 1 h. The fixed samples were then dehydrated with gradient ethanol, sputtered with carbon two times and observed by SEM (FE-SEM, Zeiss Supra).

## Results and discussion

### Interface analysis

**Figure 2:**
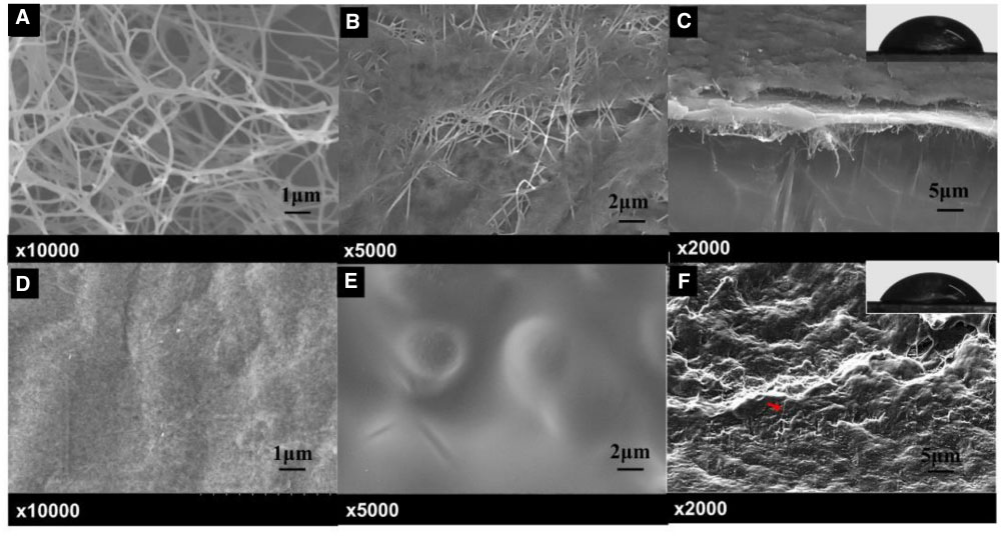
Surface and interface morphologies. (**A**) BC; (**B**) BC with WPU emulsion; (**C**) cross-section of BC−WPU; (**D**) BCG; (**E**) BCG with WPU emulsion; (**F**) cross-section of BCG−WPU; the upper right pictures of (C) and (F) were the contact angles of WPU emulsion on BC and BCG surfaces

The microstructure of surface fibrous network was observed by freeze-drying the BC and BCG. The morphology of BC showed an interconnected porous structure [40] with its nanofibers ∼200 nm in dimeter (Fig. 2A). After dropping 0.5 ml of WPU emulsion onto the BC membrane, it could be seen that the emulsion gradually covered the 3D fibrous structure of BC (Fig. 2B). As a contrast, the surface of freeze-dried BCG was very smooth with almost no nanofiber structure (Fig. 2D), because small molecule glycerol covered and penetrated into BC nanofibers; the hydrogen bonds between BC fibers may be weakened by glycerol molecular and then new and tighter hydrogen bonds between glycerol and BC fibers were formed, resulting in the collapse of the BC fibers network structure [41]. Therefore, dropped 0.5 ml of WPU emulsion onto the BCG membrane, it could be seen that WPU emulsion was directly spread to form a flat surface (Fig. 2E).

Fine wettability plays a critical role in ensuring good interfacial adhesion between two materials. Good wettability means that the liquid can spread over the entire surface of the substrate, so the degree of wettability improves with the increase of contact angle. The contact angle of WPU emulsion on the surface of BC and BCG was tested and the result was shown in Fig. 2C and F. The surface contact angles of BC and BCG were 71.6° and 66.6°, respectively, indicating that WPU emulsion had better wettability on BCG surface. Moreover, it could be seen that the interface of BC−WPU was clear with obvious BC fibers, while the interface of BCG−WPU was tight fitting without transition layer and BC fibers were difficult to identify. By comparing the microscopic morphology of the composite interface and the contact angle of WPU emulsion on BC and BCG substrates, it was found that the bonding between BCG and WPU was more compacter and firmer, which is conducive to their application as nasal stent.

### Thermal property

**Figure 3:**
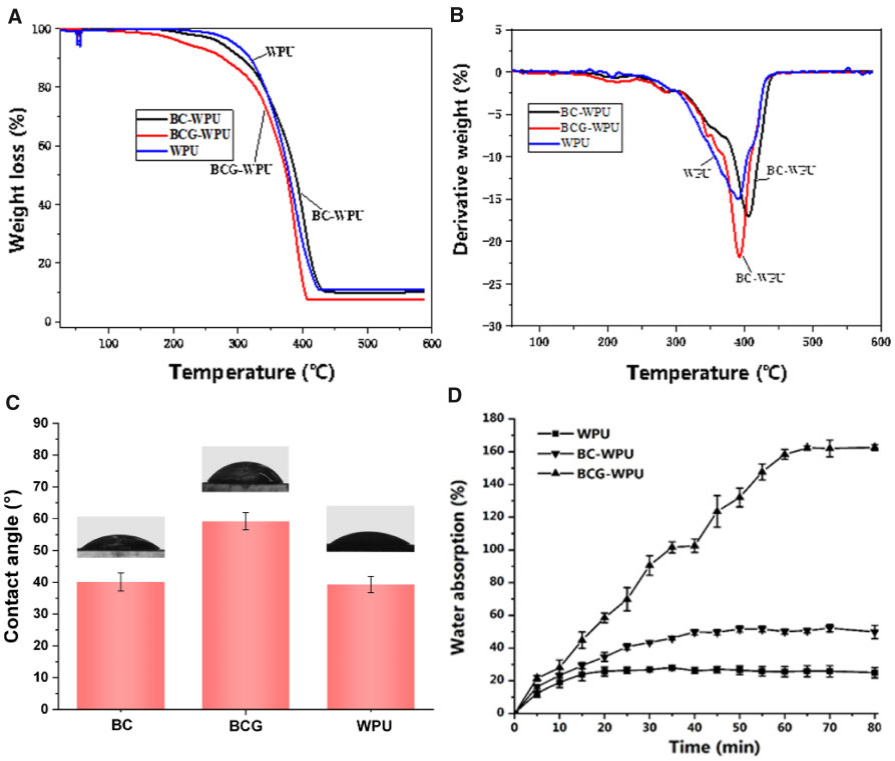
(**A**) TGA curves of WPU, BC−WPU composite and BCG−WPU composite; (**B**) DTA curves of WPU, BC−WPU composite and BCG−WPU composite; (**C**) WCA of WPU, BC and BCG; (**D**) water absorption rate of WPU, BC−WPU composite and BCG−WPU composite

The influence of BC or BCG on the thermal stability of WPU was investigated to determine the feasibility of their application as nasal stents. Figure 3A and B shows the TGA and differential thermal analysis (DTA) curves of BC−WPU, BCG−WPU and WPU. The mass loss of BC−WPU, BCG−WPU and WPU at 250°C were 5.3, 11.2 and 3.1%, respectively. At this stage, the physically adsorbed water molecules in bacterial cellulose and small molecule glycerol in BCG were volatilized. Due to the glycerol molecules infiltrated in BCG network volatilized at the beginning of thermal treatment, BCG−WPU has the highest mass loss rate at this stage. The mass loss of the BC−WPU, BCG−WPU and WPU increased rapidly around from 300 − 430°C, which was associated with the thermal degradation of WPU chains and the main cellulose skeleton. It could be seen, the thermal stability was reduced after WPU was compounded with BC and BCG, but the initial thermal decomposition of BCG−WPU was still higher than 110°C. In addition, volatilization of small molecule glycerol would not affect the use of the composites in nasal environment.

### Hydrophilicity and water absorption

The hydrophilicity could reflect relative degree of friction between medical devices and mucosa or other tissues in certain extent. WCA of WPU and its BC or BCG composites were measured to evaluate their hydrophilicities. Figure 3C shows the WCA of WPU, BC−WPU, BC, BCG−WPU and BCG. Dry BC exhibited high hydrophilicity with a small WCA of 40.1° owing to the large number of hydroxyl groups on its framework. Glycerol with a comparatively smaller hydroxyl content than BC, formed new hydrogen bonds with BC fibers, resulting in a decrease in the hydrogen bonds formed between the BC fibers. Therefore, the WCA of BCG increased to 59.2°, which means that the hydrophilicity was slightly decreased when BC was treated with glycerol. The surface hydrophilicity of WPU was rather good which is related to the large amount of hydrophilic carbamate groups, amino groups and carboxyl groups on WPU chains.

In order to improve the comfort of the patients, promote wound healing after FESS, and reduce the risk of infection, the materials used for the nasal stent are preferable to have the ability to absorbing fluid [9]. Figure 3D shows the water absorption rate of WPU, BC−WPU and BCG−WPU. The water absorption capacity of BCG−WPU and BC−WPU mainly comes from BC and BCG. WPU have reached water absorption balance after 30 min with the water absorption ratio of 26%. The BCG−WPU composites could get an equilibrium water absorption rate of 162% while BC−WPU could only reach 51% due to that BC hah lost its water absorption ability after air drying and was difficult to recover the gel state [41]. BC has high crystalline index because of many hydrogen bonds on BC fibers. Therefore, BC fibers were tightly arranged after drying in the air, which lead to the limited water absorption ability of BC−WPU. In contrast, BCG−WPU exhibited high water absorption ability, which were three times higher than that of BC−WPU. Because the glycerol molecules of BCG infiltrated into the 3D network of BC fibers and decrease the hydrogen bonding force between original BC fibers. BCG could restore the original hydrogel state after aspiration when contact with the nasal mucus in application, so as to reduce the discomfort of contacted mucous membranes, and release antibacterial drugs to promote wound healing.

### Mechanical properties

**Figure 4:**
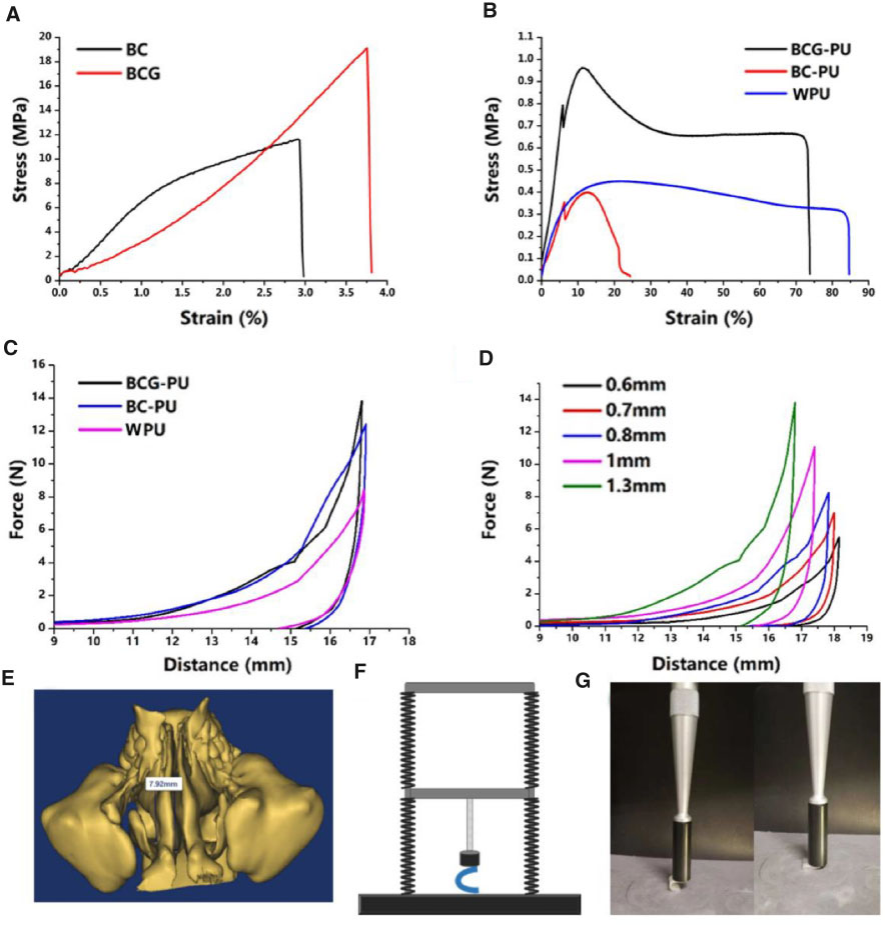
(**A**) Tensile stress−strain curves of BC and BCG (**B**); tensile stress−strain curves of BC−WPU, BCG−WPU and WPU; (**C**) the compression curves of BC−WPU, BCG−WPU and WPU at the same thickness; (**D**) the compression curves of BCG−WPU with different thickness; (E) Sinus model by MIMICS 3D reconstruction; (**F**) schematic diagram for the test of expansion supporting force; (**G**) the test samples of expansion supporting force

The stress−strain curves of BC, BCG, BC−WPU, BCG−WPU and WPU were obtained by conducting the tensile test. As shown in Fig. 4A, BC had a tensile strength of 11.63 MPa and an elongation at break of 2.91%. Both tensile strength and elongation at break of BCG were higher than pure BC because of the plasticizing treatment of glycerol. Glycerol molecules, acted as the plasticizer, can easily insert into BC network fibers to form more hydrogen bonds with BC, thus improving the tensile strength of BCG [41]. As shown in Fig. 4B, Young’s modulus and elongation at break of WPU were 4.76 MPa and 80.71%, respectively. Young’s modulus of BCG−WPU were obviously improved compared with WPU, indicating that BCG significantly improved the hardness and strength of the composite material. Both BC−WPU and BCG−WPU broke two times as shown in the stress−strain curves. BCG−WPU was broke for the first time at the elongation of 5.8% and ultimately broke at the elongation of 73.95%. The tensile strength of BCG−WPU was 0.96 MPa, and the yield tensile strength was 0.66 MPa. The tensile strength at break of BCG−WPU was almost equal to the yield strength. For BC−WPU, it quickly reached tensile strength and broke due to its high brittleness. The ultimate elongation at break of BC−WPU was only 24.5%, while BCG−WPU retained the good flexibility of WPU. The detailed data of Young’s modulus, tensile strength and elongation at break of BC, BCG, BC−WPU, BCG−WPU and WPU are shown in Table 1.

**Table 1:** Mechanical properties

	Young’s modulus (MPa)	Tensile strength (MPa)	Elongation at break (%)
BC	38.12±0.073	11.63 ± 1.93	2.91 ± 0.06
BCG	51.71±0.056	19.12 ± 0.49	3.75 ± 0.50
WPU	4.76±0.015	0.45±0.08	80.71 ± 7.43
BC−WPU	4.92±0.015	0.40±0.04	24.53 ± 1.03
BCG−WPU	12.10±0.002	0.96±0.09	73.95 ± 6.05

The nasal stent should not only resist the opposite reaction of the tissues around the ostium of the nasal sinus to open the narrow sinus cavity, but also adapt to various narrow and curved conditions in the sinus cavity, so as to conform to the sinus cavity. Therefore, it requires that the stents not only provide supporting force to enlarge the cavity of paranasal sinuses and maintain the airflow passage, but also have good longitudinal flexibility. In order to ensure that the U-shaped nasal stent can support the sinus mucosa and keep the sinus unobstructed without causing too much pressure on sinus canal to hinder wound healing, we designed a U-shape compression experiment to test the supporting force of the stent with different thickness and materials. The diameter of human nasal passage measured by MIMICS was 7.92 mm, so the U-shaped stent in human nasal cavity had an expansion of ∼8 mm. On the compression curve of the stent, the force of stent expanded to 8 mm was selected to represent the expansion supporting force. Figure 4F shows the schematic diagram for the test of supporting force. Figure 4G shows the test process of expansion supporting force. The compression curves of BC−WPU, BCG−WPU and WPU at the same thickness are shown in Fig. 4C. As shown in Table 2, WPU exhibited the lowest expansion supporting force of 0.57N. On the compression curves of BCG−WPU and BC−WPU, BC and BCG layer broke first when the samples were bent too much, leaving turning points on the curves. The supporting force of BCG−WPU and BC−WPU was larger than that of WPU at the same thickness, but BC−WPU exhibited poor tensile properties as mentioned above. Therefore, BCG−WPU was chosen for further investigation on support forces of materials with different thicknesses, and the results are shown in Fig. 4D. The expanding supporting force of U-shape samples at the expansion degree of 8 mm is shown in Table 3. It could be seen that the expanding supporting force of BCG−WPU raised with increasing the thickness of the composites. In the sinus, the supporting force should not be too large, otherwise the mucosa would be damaged. The size and thickness of the sinus stent can be selected to ensure the effect of the stent through the measurement of the expansion supporting force.

**Table 2:** The maximum supporting force and the expansion supporting force of WPU, BC−WPU and BCG−WPU

Sample	WPU	BC−WPU	BCG−WPU
Maximum supporting force/N	8.43	12.41	13.82
Expansion supporting force/N	0.57	1.14	1.22

**Table 3:** U-shape supporting force of BCG−WPU with different thickness

Thickness/mm	0.6	0.7	0.8	1	1.3
Support force /N	0.36	0.59	0.86	1.22	1.36

Figure 4E shows a 3D reconstruction model of the MMIMIC of the human facial sinus structure. Among them, the ethmoid sinus was located in the upper part of the nasal cavity and composed of gas-filled small houses with different degrees of gasification. Each side had 3 − 18 small gas rooms. The size, arrangement and extension range of the gas room were extremely irregular, and the two sides were extremely asymmetrical. The ethmoid sinus has a complex structure, therefore it was difficult to clearly observe its structure when utilizing other methods, which made it difficult for undergoing surgery and treatment. The small air chamber of the ethmoid sinus and the full view of sinus structure could be clearly observed with the MIMICS, which improved the accuracy and shorten the analysis time. The size and morphology of each part of sinus could be visually observed and measured by using the MIMICS 3D reconstruction function. Nasal stents for patients with special nasal structures could also be customized by MIMICS measurements. The actual size of the sinus could also be measured by MMIMS to provide guidance for the size of the stent.

### Antibacterial property

**Figure 5:**
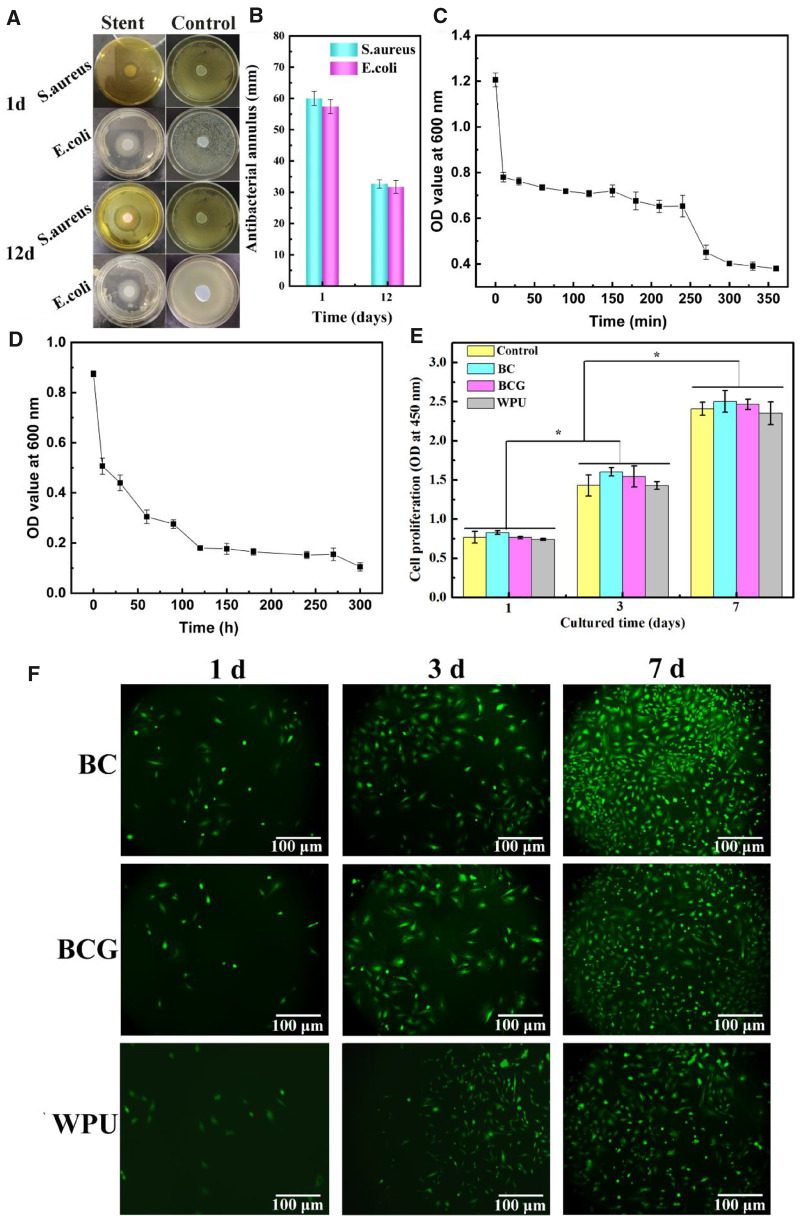
The inhibition zones (**A**) and their corresponding size (**B**) of the drug-loaded BCG-PU composite against *S. aureus* and *E. coli*; (**C**) OD values of BCG drug-loaded samples cultured with *E. coli* solution at different release time points; (**D**) OD value of WPU drug-loaded samples cultured with *E. coli* solution at different release time points; (**E**) OD values of control and experimental groups of L929s after 1, 3 and 7 days of culture; (**F**) the fluorescence images of living cells on BC, BCG and WPU at 1, 3 and 7 days of culture with Calcein-AM staining

In order to prevent infection and inflammation after surgery, the PHMB was loaded onto the BCG−WPU and tested the antimicrobial properties of the composite. The size of the inhibition zone of the drug-loaded composite and its antibacterial efficiency against *S. aureus* and *E. coli* are shown in Fig. 5A and B. The size of the inhibition zone of the drug-loaded stent against *S. aureus* and *E. coli* on the first day were 60.0 ± 2.3 and 57.4 ± 2.1 mm, respectively. After 12 days, the stent still had favorable antibacterial properties. The size of the inhibition zone of the drug-loaded stent against *S. aureus* and *E. coli* on the 12th day were 32.7 ± 1.3 and 31.7 ± 2.1 mm, respectively. Since BCG and WPU have no antibacterial properties, the size of the inhibition zone of the control group against *S. aureus* and *E. coli* on the 1st and 12th day was 0 [42].

Figure 5C and D shows the OD value of BCG and WPU drug-loaded samples cultured with *E. coli* solution at different release time points. As shown in Fig. 5C, the OD value of control was 1.206, and the OD value of BCG drug-loaded sample shaking for 10 min decreased to 0.78. With the prolongation of oscillation time, PHMB in BCG was released continuously and reached equilibrium at ∼6 h. From Fig. 5D, the extracts of WPU drug-loaded membrane with 12 days of oscillation still had obvious antibacterial activity. The antibacterial effect curves of BCG and WPU drug-loaded samples indicated that the BCG drug-loaded membrane could achieve antibacterial activity in a short time with remarkable antibacterial effect. The WPU drug-loading membrane could maintain the antibacterial activity for a long time because the antibacterial agent was difficult to release quickly. Therefore, BCG−WPU double-layer nasal stent loaded with PHMB can not only achieve short-term and efficient anti-inflammatory effect post-operation, but also continuously and steadily release drugs in it, which played a continuous antibacterial role in the process of wound healing.

### *In vitro* cytocompatibility

The CCK-8 assay and L929s cultured on its surface were carried out to evaluate the biocompatibility and biosafety of BC, BCG and WPU. The results of CCK-8 assay are shown in Fig. 5E. The OD value is proportional to the number of viable cells. With the culture time increasing from 1 to 7 days, the viability of cells in sample extracts showed a significant increase. Compared with the control group, the relative proliferation rate of BC, BCG and WPU was more than 95%. It can be concluded that the extracts of WPU, BC and BCG have no significant cytotoxicity.

L929s were cultured on the surface of BC, BCG and WPU to assess the cell biocompatibility. It can be seen that L929s on all samples exhibited spindle or polygonal morphology. On the first day of culture, the surface of WPU adhered less L929s than BC and BCG which may be due to that its smooth surface was not conductive to cell adhesion. On the seventh day of culture, a large number of cells proliferated on the surface of BC, BCG and WPU, evenly distributed and formed a monolayer. There was no significant difference of cell number and morphology between BC and BCG surface, indicating that plasticizing did not affect the cytocompatibility of BC.

### Performance in the rabbit’s nasal cavity

**Figure 6:**
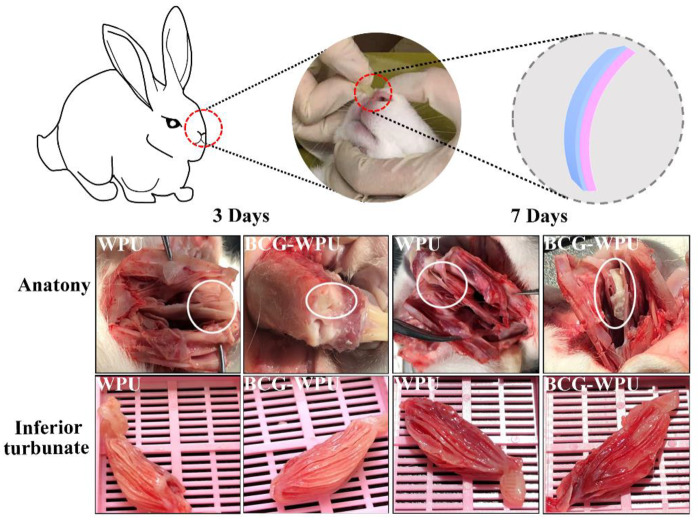
The diagram of implantation process and anatomy after implantation for 3 and 7 days

Bernhard *et al*. [43] tested the mechanical properties of the nasal mucosa dissected from the corpse. They have arrived the elongation at break and the tensile strength of the nasal mucosa with the size of 10 × 20 × 0.1 mm were 28.6% and 5.9 MPa, respectively. The supporting force in the sinus should not be too large, otherwise it will cause damage to the mucosa. Therefore, we calculated the tensile force of 1% of the deformation rate of the nasal mucosa, and found that the thickness of the stent that meets the conditions of the nasal cavity support force was 0.7 mm. Therefore, the samples of BC−WPU loaded with PHMB with the size of 1.2 × 0.5 × 0.7 mm were used for the *in vivo* study. The purpose of *in vivo* study was to evaluate the biocompatibility and the histological response of the samples which contacted with the inferior turbinate mucosa of rabbits. The anatomical results of rabbits on the third and seventh days are shown in Fig. 6. The volume and color of the inferior turbinates were normal on the third and seventh day. All of the samples taken out were intact on the third day, and a small amount of nasal secretion adhered to the surface of the samples; On the seventh day, the surface of WPU samples was slightly wrinkled and a little white nasal secretion adhered to the surface of the samples.

**Figure 7:**
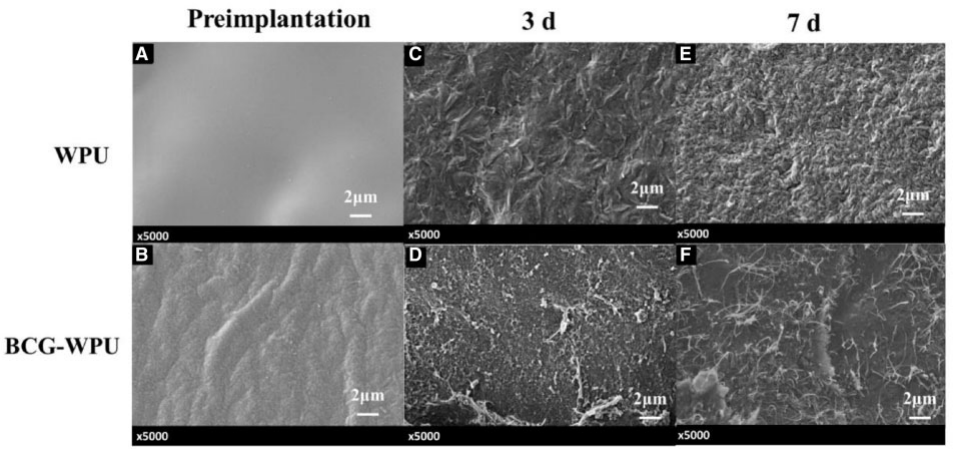
The surfaces morphology of WPU and BCG−WPU composite before and after 3 and 7 days of implantation

The surface SEM images of the WPU and BCG−WPU before and after implantation were shown in Fig. 7. It could be seen that the surface of WPU was flat and slippery before implantation (Fig. 7A), while slight wrinkles appeared on the surface of WPU after third day of implanting (Fig. 7C). The uneven surface of WPU after 7 days of implanting may be due to the degradation of the surface layer of WPU membrane and the adhesion of nasal secretions and bacteria (Fig. 7E). As shown in Fig. 7B, the surface of BCG−WPU was not smooth, but not yet obviously rough. When the BCG−WPU samples were implanted for 3 days, except of some adherents, no significant change was observed on the BCG surface (Fig. 7D). The cluttered nanofibers can be seen on the surface of BCG−WPU sample after 7 days of implantation, which may be due to that the BCG layer became gelatinous after absorbing liquid in the nasal cavity. The bacterial cellulose swelled and retained its 3D nanofibrous network. The gelation of BCG layer was beneficial to the release of antimicrobial agents and the reduction of nasal mucosa injury, which could contribute to the wound healing of nasal after surgical operation.

**Figure 8:**
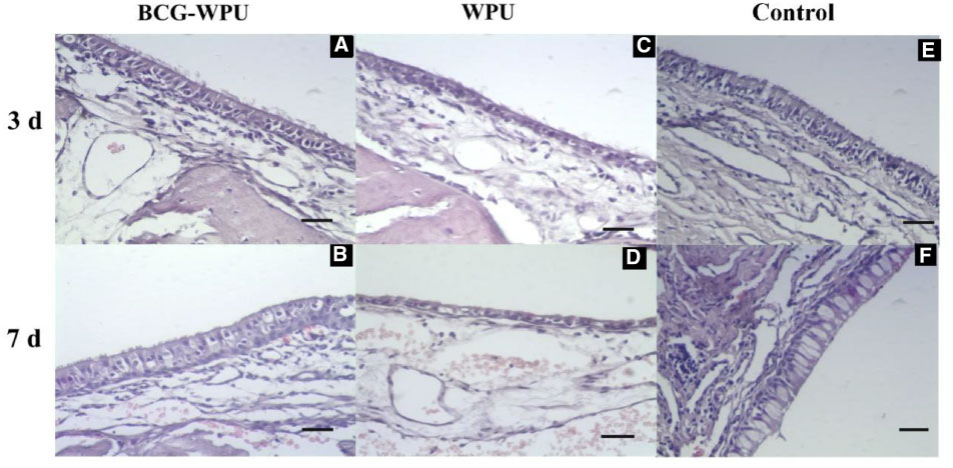
Histologic staining of the inferior turbinate mucosa after the placement of WPU and BCG−WPU composite. Scale bar: 50 μm

The main effects of the implants to the nasal mucosa were damages to the mucosal epithelial tissue and the local inflammatory reaction. Figure 8 shows the histologic staining images of the inferior turbinate mucosa after the placement of WPU and BCG−WPU. For BCG−WPU samples implanted for 3 days, the cilia of the epithelial cells of the inferior turbinate were partially shed, and mild neutrophil infiltration and mild epithelial cell shrinkage occurred (Fig. 8A). There was no significant change in neutrophil infiltration, and the shrinkage of epithelial cells was slightly aggravated after implanted for 7 days. And as a side benefit, the goblet cells and the glandular epithelial cells increased at seventh day of implantation (Fig. 8B). For WPU samples implanted for 3 days, the epithelial cells of the inferior turbinate were shed and shrunk, the epithelial space was enlarged, and mild neutrophil infiltration occurred (Fig. 8C). There was no significant change in neutrophil infiltration after implanted WPU for 7 days. But the epithelial cell shrinkage was significantly aggravated and the epithelial space was significantly enlarged, which may be due to the fact that hard materials are not conductive to epithelialization (Fig. 8D). In the control group, there were no stents implanted in the nasal cavity, so the nasal mucosa was intact and no cilia of the epithelial cell fell off (Fig. 8E and F). Therefore, it could be concluded that the BCG−WPU composite had less damage to the nasal mucosa, mainly due to the gelation of BCG layer protecting the nasal mucosa after absorbing the nasal mucus. The BCG−WPU double-layer nasal stent could simultaneously reach the effectiveness of both supporting the nasal cavity and protecting the surgical wound of the nasal cavity, which achieved the initial intention of designing the bifunctional nasal stent.

## Conclusion

In this paper, plasticized bacterial cellulose (BCG) and WPU composite with antibacterial function used as a novel nasal stent was first designed and prepared. Specifically, due to the gelation of BCG after absorption liquid, BCG contacting with nasal mucosa could create a moist environment to promoting would healing. Moreover, the strength of WPU was significantly improved by composited with BCG, which could provide sufficient toughness and tension for the prefabricated space. The cytotoxicity test showed that both the WPU and the BCG were non-toxic, and fibroblasts cultured on the surface of WPU and BCG revealed that the L929 cells exhibited normal growth and proliferation. Antibacterial performance measurements of the BCG−WPU composite loaded PHMB demonstrated that the composite maintained good antibacterial effects for at least 12 days. Animal experiments further confirmed that both WPU and BCG−WPU composite would not cause serious infection and inflammatory after implantation into the nasal cavity of rabbits, while BCG−WPU composite had less damage to the mucosal epithelium. The BCG−WPU drug-loaded composite used as a nasal stent could not only support the nasal cavity, but also protect the wound and promote wound healing owing to the gelation of BCG. Additionally, accurate and long-lasting release of antimicrobial agents may improve the medication safety of patients. In conclusion, we believe that the BCG−WPU drug-loaded composite used as nasal stent proposed in this study can provide a new treatment option for patients with nasal diseases.
